# Pre‐operative prediction of soft tissue balancing in knee arthoplasty part 1: Effect of surgical parameters during level walking

**DOI:** 10.1002/jor.24289

**Published:** 2019-04-12

**Authors:** Marco Viceconti, Daniele Ascani, Claudia Mazzà

**Affiliations:** ^1^ Department of Industrial Engineering University of Bologna Viale Risorgimento 2 Bologna 40136 Italy; ^2^ Laboratorio di Tecnologia Medica IRCCS Istituto Ortopedico Rizzoli Bologna Italy; ^3^ Department of Mechanical Engineering and INSIGNEO Institute for in silico Medicine University of Sheffield Sheffield United Kingdom

**Keywords:** total knee arthroplasty, soft‐tissue balance, patient‐specific model

## Abstract

An important reason for poor functional outcome of Total Knee Arthroplasty is inadequate soft tissue balancing. Custom‐made cutting guides or computer‐aided surgical navigation make possible to accurately achieve what is planned; the challenge is to perform a pre‐operative planning that properly accounts for soft‐tissue balancing. The first step in the development of a patient‐specific computer model that can predict during pre‐operative planning the post‐operative soft‐tissue balancing is a better understanding of the role that cutting heights and angles have on the balancing of the soft tissues after TKA as the patient perform the more common daily tasks. In the present study, we conducted a sensitivity analysis of the ligament elongations during level walking due to TKA as a function of position and orientation of the cutting guides, by means of a validated patient‐specific dynamic model of the post‐TKA knee biomechanics. The results suggest a considerable sensitivity of the collateral ligaments elongation to the surgical variables, and in particular to the varus‐valgus angles of both tibia and femur. This complete elongation map can be used as a baseline for the development of reduced‐order models to be integrated in pre‐operative planning environments. © 2019 The Authors *Journal of Orthopaedic Research*. Published by Wiley Periodicals, Inc. J Orthop Res 37:1537–1545, 2019.

Total Knee Arthroplasty (TKA) is a surgical procedure aimed to replace the damaged surfaces of the knee joint with artificial components. The increasing number of osteoarthritis patients due to the progressive aging of the population boosted the demand of total knee replacement surgeries, which now present in most clinical studies a success rate of 90% or better, when measured in term of revisions.^1^, ^2^, ^3^, ^4^ However, some studies report that over 40% of patients are unsatisfied with the life style that their TKA offers.^5^ This is expected to get worse as younger, more physically active patients go through surgery (a group that already accounts for 45% of TKA patients, according to ref.^6^). In fact, younger patients that have higher expectations in term of active life style, are not satisfied with their prosthesis and more prone to revision surgeries.^7^ Many of these functional complications after TKA are caused by a non‐optimal balancing of the residual knee ligaments left by the surgery.^8^, ^9^

Achieving an optimal balance between stability and mobility requires both an accurate pre‐operative planning (to ensure an optimal balancing of the soft tissues), and an accurate execution (to accurately achieve the planned skeletal positioning). In principle, both computer‐aided surgical navigation and patient‐specific instrumentation such as custom‐made cutting guides should ensure accurate execution, but in spite of this both technologies have contradictory reports in terms of clinical outcome.^10^, ^11^, ^12^, ^13^, ^14^, ^15^, ^16^, ^17^, ^18^, ^19^ One major limitation is that for all these technologies, pre‐operative planning is performed on static models that provide detailed anatomical information but no functional information. If pre‐operative planning technologies could provide a reliable prediction of the functional outcome of TKA, it is reasonable to expect these technologies would be much more effective.

In principle, patient‐specific inverse multibody dynamics computer models could be used for this purpose. However, such models require as input the full 3D kinematics of the movement. Even if pre‐operative acquisition of the patient's gait was routinely possible, since patients compensate post‐operatively for any functional alteration by adjusting their kinematics and kinetics, such measurements would not be informative. Thus, the pre‐operative prediction of the functional outcome must necessarily rely on reduced‐order models that do not need full kinematics as input.

A first step toward the development of such reduced‐order models is the better understanding of the role that cutting heights and angles have on the balancing of the soft tissues after TKA as the patient perform the more common daily tasks. Such investigation, conducted on a TKA patient once the rehabilitation is completed and the post‐operative neuromuscular control during level waking has stabilized, would provide a baseline accuracy against which any reduced‐order models could be compared. Such sensitivity analysis could not be conducted experimentally, as the same patient cannot be operated multiple times; however, this would be possible using a validated patient‐specific computer model of the musculoskeletal dynamics during level waking.

The aim of the present study is to conduct a sensitivity analysis of the ligament elongations during level walking due to TKA as a function of position and orientation of the cutting guides, by means of a validated patient‐specific dynamic model of the post‐TKA knee biomechanics.

## MATERIALS AND METHODS

### Experimental Data for Dynamics Model

The experimental data used in this study come from the third “Grand Challenge Competition to Predict In Vivo Knee Loads” data,^1^^,^ 20, 21 because of the availability of a pre‐operative MRI exam in this particular data collection. Institutional review board approval was obtained, and the subjects gave informed consent for data collection and distribution.^20^

The data were obtained from a female subject (height = 167 cm, BW = 78.4 kg), who received in her left knee a posterior cruciate‐retaining total knee replacement prosthesis (*eTibia*) capable of recording and transmitting via telemetry the instantaneous knee resultant force. The dataset includes pre‐operative MRI, post‐operative CT, geometries of the lower limb bones and of the total knee prosthesis, strength data measured with a BIODEX isokinetic dynamometer, motion analysis data for level‐walking including EMG signals from 15 lower‐limb muscles, and the *eTibia* recordings, synchronized with gait and EMG data. The six‐component generalized force vector the eTibia provide can be decomposed in two compartmental force *F*_medial_ and *F*_lateral_, using a simple regression equation.

### Subject‐Specific Musculoskeletal Model

**Figure 1:**
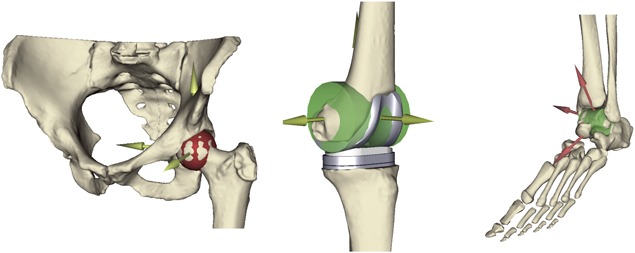
The definition of the body joints: Ball socket (hip) and hinge (knee and ankle).

The experimental data made available in the 3rd Knee Grand Challenge^21^ where used to generate a subject‐specific model of the lower limb dynamics, following the methods described in ref.^22^ The five provided bone geometries (pelvis, femur, patella, shank, foot) and two additional ones (metatarsal and toe) derived from another model^23^ and scaled, were aligned using the NMSBuilder open source software.^24^ Inertial properties were assigned to each segment according to ref.^25^ The hip joint was idealized with a ball‐and‐socket, while the knee and ankle joints were idealized as hinges. A least square algorithm (Matlab, MathWorks, USA) was used to automatically fit a sphere to the femoral head, and a cylinder to the distal femur and talus trochlea^22^; the algorithm relies on the LSGE Matlab Library (NPL Centre for Mathematics and Scientific Computing, UK). When the post‐operative knee was modeled, the axis of ration of the hinge was based on the implant design. While the specific implant had a double radii design, since during most of the waking cycle the posterior part of the femoral prosthetic implant was in contact with the polyethylene insert of the tibia component that curvature was used to define the rotational axis (Fig. 1).

The patella‐femoral joint was modeled with a custom joint, where the frontal and transversal rotations were neglected to describe the movement of the patella on the femur. The motion path was described considering the congruency of the patella button with the surface of the femoral component, which can be accurately described as an arc of circle. The motion of the joint then was defined using a spline where the four degrees of freedom were coupled with the knee joint angle. This constraint allowed having a correct movement of the patella entirely dependent by the knee flexion–extension angle. The talus trochlea joint was defined as a hinge joint in the same manner, fitting a cylinder to the bone and describing the rotation axes of the ankle flexion–extension.

**Figure 2:**
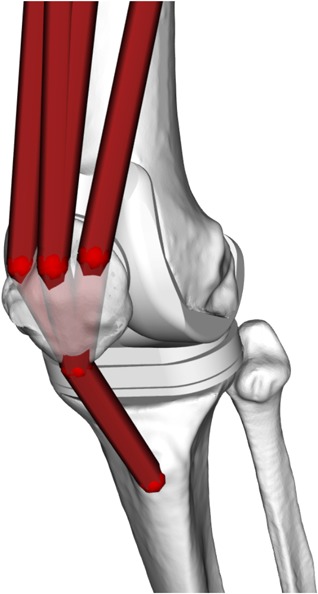
The musculoskeletal model was modified to transmit the forces of the quadriceps through the patella to the tibia.

An atlas of 43 Hill‐type musculotendon units^23^ was scaled to the skeletal geometry using an affine transformation based on well‐defined skeletal landmarks,^26^ again with the NMSBuilder software. The atlas included also via‐points to define the correct wrapping path for selected muscles. Quadricep muscles were wrapped around the patella and attached to the tibia through the patellar ligament insertion. Mechanically, the quadriceps muscle forces were transmitted along the line of action of the patellar ligament and the patella body worked as frictionless pulley during the knee flexion. This mechanism allowed to estimate the correct knee contact forces^27^ (Fig. 2).

The Lateral Collateral Ligament (LCL) and the Medial Collateral Ligament (MCL) were included in the lower limb model. The estimation of the ligaments origins and insertions were computed on the preoperative CT scan following a validated procedure.^28^ The affine registration allowed the registration of the ligaments’ attachments on the patient's bone geometries along with a medial point that permits the wrapping of the MCL around the femur and tibia, imitating the anatomical path on the bone surface. Hence, the LCL was represented as a straight line whilst the MCL is composed by two connected line segments. All the 3D elements of the subject‐specific model were inspected by superimposing them to the CT and/or MRI images, and a visual inspection performed by an expert operator suggested that the matching was very accurate.

Knee ligaments were modeled as one‐bundle non‐linear springs, according to ref.^29^ Ligament parameters (stiffness, reference strain, and resting length) were derived from the literature.^30^ Muscle parameters were derived from ref.^23^, except those of the quadriceps (whose insertion was moved from the patella to the tibial tuberosity), which were extracted from DeMers et al.^31^

Last, in order to replace the dynamic contribution related to the missing torso and contralateral leg, coordinate actuators acting to the 6 degrees of freedom of the pelvis respect to the ground were added to the model.

The resulting model was used to track four separate gait cycles recorded experimentally, using a standard global optimization algorithm.^15^ For each gait cycle, a complete inverse dynamics solution was computed to balance the recoded ground reaction. Last, static optimization was used to compute muscle forces, assuming as cost function the sum of the squares of the muscle stresses. All calculations were performed using OpenSim v3.3 (NCSSR, USA).

**Figure 3:**
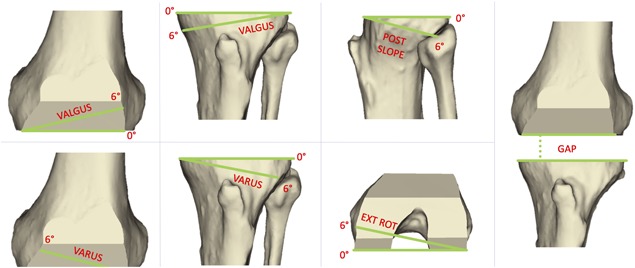
Surgical variables (from left): varus‐valgus femur, varus‐valgus tibia, tibial slope, external rotation femur. The fifth variable (gap) is the distance between the two distal cutting planes of femur and tibia.

### Verification and Validation

Once the patient‐specific model was completed, we conducted a systematic verification and validation study on it. The OpenSim software is extensively benchmarked (Seth, 2018). The inverse dynamics solution of our model was verified for conservation of momentum (RMSE < 0.1%). A number of previous studies showed for very similar models the moderate sensitivity to inputs uncertainty.^32^, ^33^, ^34^, ^35^ As validation, the knee contact forces predicted by the model were compared to the experimental values recorded by the instrumented implant. The predictions of the four walking gait trials were expressed as a fraction of the BW and resampled on a 0–100% trial duration scale with a step interval of 1% from heel strike to the subsequent heel strike. The differences between model prediction and experimental data were quantified in terms of magnitude, RMSE, and coefficient of determination (*R* ^2^). The computed knee joint reaction forces were also compared using the magnitude and the timing of their two typical main peaks and the similarity of their shape.

### Sensitivity of Soft Tissue Balance to the Surgical Variables

Once the biomechanical alignment with the hip and ankle centers is achieved,^36^ the following surgical parameters are usually varied to define the actual positioning of the knee prosthesis (Fig. 3):

1. The orientation in the frontal plane of the varus‐valgus femur cutting plane;
2. the orientation in the frontal plane of the varus‐valgus tibia cutting plane;
3. the orientation in the frontal plane of the internal‐external rotation femur cutting plane;
4. the orientation in the sagittal plane of the posterior slope of the tibial cutting plane;
5. gap between the femoral and tibial cutting planes.

Solving repeatedly the model each time modeling different values for these surgical variables allowed a systematic exploration of how sensitive the elongation of the knee collateral ligaments is to changes in the gap distance and in orientation of the cutting planes. The following values where used:

1. Varus‐valgus femur cutting plane: From −3° to 3°, with a step of 1°;
2. Varus‐valgus tibia cutting plane: From −3° to 3°, with a step of 1°;
3. External rotation femur condyle cutting plane: From 0° to 6°, with a step of 1°;
4. Posterior slope tibial cutting plane: From 3° to 5°, with a step of 1°;
5. Gap between cut planes: From 18 to 28 mm, with a step of 2 mm.

These surgical parameters are those most commonly controlled by the surgeons in various TKA pre‐operative planning tools to define the position of the femoral and tibial components on the bones. A sensitivity analysis was conducted throughout four normal gait cycles, by varying each surgical parameter independently.

In the patient under examination here, the postoperative geometries of the bones were already shaped to simulate the actual TKA surgery and the prosthetic implants were already placed on the subject. The surgical procedure adopted comprised one cut on the tibia and five cuts on the femur: (1) distal cut (2) anterior and posterior femur cuts (3) anterior and posterior chamfer cuts.^37^ The description of the surgical procedure employed and the intraoperative details for this subject were not provided, thus we assumed that all the surgical variables of the preoperative preplanning were in the neutral position (all the parameters set at 0° except the posterior slope at 3°) and the same condition was preserved after the surgery.

**Figure 4:**
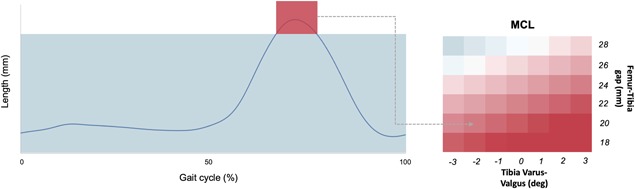
Example of the heat map construction. On the left the curve of the length of the MCL ligaments throughout the gait cycle with a Tibia Varus‐Valgus = −2° and gap = 26 mm. On the right the heat map of the Tibia Varus Valgus parameter. Red color = 10% of the initial length, Blue color = −10% of the initial length.

A number of studies^38^, ^39^, ^40^ on human cruciate ligaments suggest an elongation to failure in the range of 15–19%, and no irreversible effects for elongations found for values lower than 10%; this threshold value was thus assumed in this study. The representation of the results of the sensitivity analysis was performed using a heat map for each surgical variable. For each combination of surgical parameter, for each of the four gait cycles, and for each of the two ligaments the simulation produced an elongation plot like the one in Figure 4 (left). To simplify the analysis, only the peak elongation was considered. The heatmap color scale was set to go from dark aquamarine for strain equal or lower than −10% (slack) to dark red for strain equal or higher than +10% (elongation); white indicated zero strain.

## RESULTS

### Model's Validation

**Figure 5:**
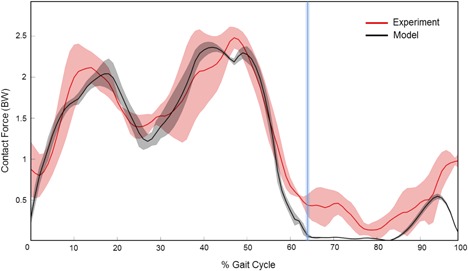
Total knee joint forces predicted (black) during four walking gait trials normalized on the 100% of the gait cycle. The eTibia experimental forces are showed for the same gait trials (red). The vertical blue bar represents the toe‐off phase of the gait cycle (toes are leaving the ground).

The knee contact force predicted by our model and that measured by the instrumented implant over the four gait cycles and similar in amplitude and timing (Fig. 5).

The total force measured by the instrumented prosthesis reported two peaks throughout the full gait cycle, with the first peak of 2.0 BW occurring at the beginning of the stance, and a second peak of 2.6 BW occurring toward the end of the gait cycle. The model showed an excellent accuracy showing a difference of 0.1 BW when compared to the prosthesis data for both peaks (Table 1).

**Table 1:** Joint Contact Forces Measurements in a Patient With Implanted Instrumented Prosthesis Compared to Predicted Model From a Subject Specific Model of the Same Patient

	1st Peak		2nd Peak	
	Experiment	Model	Experiment	Model
Magnitude (BW)	2.0 (0.1)	2.1 (0.1)	2.6 (0.1)	2.5 (0.1)
Timing (% gait cycle)	13 (3)	17 (2)	48 (5)	44 (4)

The timing was also found in good agreement, as the predicted peak force was shifted by less than 4% of the gait cycle duration, from the measured one. The computed joint contact forces were highly correlated to those measured experimentally (*R* ^2^ = 0.88, *p* < 0.01), with a Root Mean Square Error (RMSE) of 0.35 BW ± 0.05 BW.

### Sensitivity of Soft Tissue Balancing

In the actual position of the knee implant, the model predicted an average MCL force of 45 N, and a peak value of 60 N, reached at around 75% of the gait cycle, when the knee is approximately at 60° flexion. The force through the LCL was even smaller, and never exceeded the 10 N.

The pre‐operative length of the knee collateral ligaments was found to be 90.5 mm and 59.4 mm for the MCL and LCL, respectively. The post‐operative length was 97.3 mm (+8% longer than preoperative length) and 60.4 mm (+2% longer than preoperative length). The gap between the femoral cut and the tibial cut in the neutral postoperative position, considered as the thickness of the prosthetic implant, was 26 mm.

**Figure 6:**
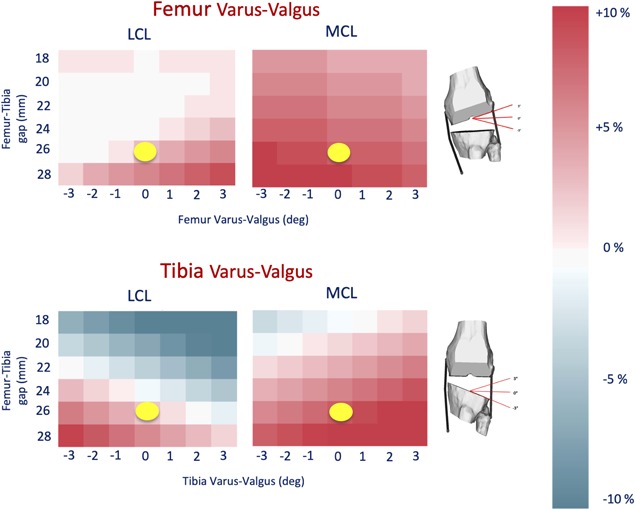
The figure shows the results of the Femur and Tibia varus‐valgus parameters. The yellow dot represents the actual surgical parameter values achieved in the surgery.

**Figure 7:**
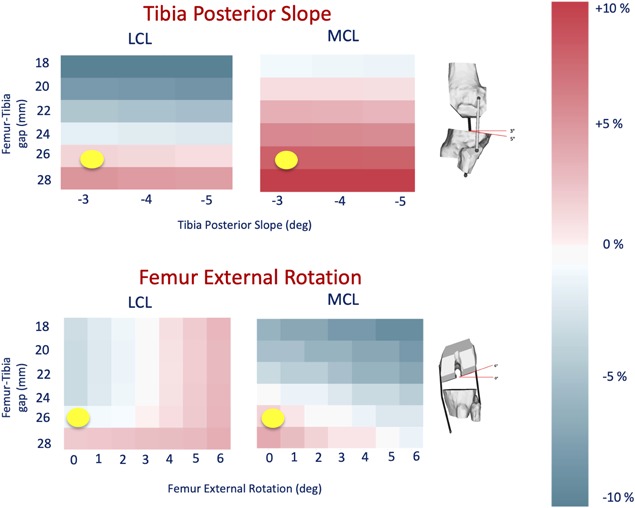
The figure shows the results of the Femur External Rotation and Tibia Posterior Slope parameters. The yellow dot represents the actual surgical parameter values achieved in the surgery.

When the surgical variables were modified, the pre‐operative length of the two collateral ligaments changed considerably. The full sensitivity to femur and tibia varus‐valgus as a function of the gap is reported in Figure 6, while Figure 7 shows the effect of tibial posterior slope and femoral external rotation, again as function of the gap.

As expected the post‐operative elongation of collateral ligaments is quite sensitive to the varus‐valgus angle. The MCL is most sensitive, and any gap of 26 mm or greater stretches it beyond the 10% limit. In comparison, the LCL seems less sensitive, reaching the limit only for a gap of 28 mm and a varus angle of 3°. The effect of tibial slope and femoral rotation was less marked, and the MCL reached the limit only for gaps of 28 mm.

## DISCUSSION

The aim of the present study was to conduct a sensitivity analysis of the knee ligament elongations during level walking due to TKA as a function of position and orientation of the cutting guides, by means of a validated patient‐specific dynamic model of the post‐TKA knee biomechanics.

The patient‐specific model predicted with good accuracy (RMSE = 270 N; *R* ^2^ = 0.88) the knee contact forces measured during level walking. The 2012 Grand Challenge, where the data of the patient we analyzed here we used, had two winners.^21^ If we look at the average accuracy across two gait trials, for the total knee contact force, the blinded prediction (RMSE = 372 N; *R* ^2^ = 0.82) and the unblinded prediction (RMSE = 280 N; *R* ^2^ = 0.88) have accuracies very close to those reported here. A crude error propagation analysis we made assuming static equilibrium suggests that this uncertainty in the knee contact force can bias the prediction of ligaments elongation of less than 1%, making the results of this study clinically relevant.

The predicted ligament forces compared well with other values found in the literature.^41^

The results on the post‐operative elongation suggest a considerable sensitivity of the collateral ligaments elongation to the surgical variables, and in particular to the varus‐valgus angles of both tibia and femur. These findings are in good agreement with those reported in a recent cadaveric study.^42^ In the specific case we analyzed, better soft tissue balancing would have been achieved with a smaller gap, and a bit more varus orientation, to shorten the MCL. While this knowledge a posteriori is not useful for the individual patient, the complete elongations map can be used as a baseline for the development of reduced‐order models to be integrated in pre‐operative planning environments, which is the long‐term goal of this research.

A number of idealizations were made to build the patient‐specific model. Among others, infinitely rigid bones, idealized frictionless joints with infinite stiffness in the unconstrained directions, lumped inertial properties, musculotendinous units modeled as Hill‐type 1D actuators, optimal motor control (minimization of the summation of muscle stresses squared). The effect of all these is accounted in the predictive accuracy of the model, in this particular case expressed in term of accuracy in predicting the knee contact forces measured by the instrumented implant. But probably the most important limitation derives from the assumption that in no case the surgery would alter the gait kinematics; in other words, we assume the compensation strategies are effective in retaining a “normal” kinematics after surgery. Of course, this is true up to a point, and for some extreme value in the rage of surgical parameters explored this is quite unlikely.

A more specific limitation is that we assumed that the balance of all soft tissues wrapping the implanted knee could be reduced to the balance of the two collateral ligaments. The method we used here work very well also for the cruciate ligaments, when the implant design preserves any (which is not the case for implant examined here). In principle, the method we used^28^ to model the patient‐specific anatomy of the ligament insertions could be extended to include also other soft tissue bundles; but the implicit assumption here is that the ligaments are much stiffer than any other connective soft tissue wrapping the knee, and damage at lower strain. Thus, it is reasonable to assume any functional limitation linked to soft tissue balancing would raise first and foremost from them.

A more structural limitation of this study is that we did not focus our attention of the effect surgical variables on the patellar tracking and the elongation of the patellar tendon, which is a well‐known functional complication for TKA. While the methods used here could in principle be used also to explore this aspect, this was considered beyond the scope of this study.

All the conclusions we reached in this study are valid only for this patient, and cannot be extended to any population, without further studies on a much larger cohort.

The method used in this study, which can be implemented whenever gait analysis and imaging data are available, can be used to evaluate the post‐operative soft tissue balance while performing daily life activities such as level walking. This could be useful, for example, for a patient with functional limitations to understand if these can be attributed to ineffective soft tissue balancing.

Because of delicate balance between kinematics, dynamics, and soft tissue balancing, this detailed analysis is possible only post‐operatively, when the actual functional kinematics can be measured on the individual patient. If soft tissue balancing needs to be predicted during walking pre‐operatively, reduced‐order models that do not require a detailed lower limb kinematics as input need to be developed. This study can provide an excellent baseline to estimate the impact that order reduction can have on the predictive accuracy of the new solutions.

In conclusion the elongation of the knee ligaments was found to be sensitive to the surgical parameters that define the positioning of the femoral and tibial components. The elongations were computed within a full‐order dynamic simulation, which accounted for all forces transmitted, and that could be validated because of the availability of telemetric knee force recordings. As such they provide an ideal baseline for the development and validation of reduced‐order models to be embedded in surgical planning simulators.

## AUTHORS' CONTRIBUTIONS

Marco Viceconti wrote this paper and supervised the PhD project from which this works derives. Daniele Ascani was the PhD student who run that project and did most of the modelling work and revised the manuscript. Claudia Mazzà co‐supervised the project and revised the manuscript. All authors have read and approved the final submitted manuscript.

## Endnotes

1 Data available at the SimTk.org website https://simtk.org/home/kneeloads.
